# Automated radiosynthesis of 2-[^18^F]BPA for PET-based planning of boron neutron capture therapy (BNCT): rational precursor design, radiofluorination, and characterization of methodology

**DOI:** 10.1186/s41181-026-00436-0

**Published:** 2026-03-05

**Authors:** Vincenzo Paolillo, Cong-Dat Pham, Robert Ta, Dimitra K. Georgiou, Henry Charles Manning

**Affiliations:** 1 Cyclotron Radiochemistry Facility, The University of Texas MD Anderson Cancer Center, Houston, TX USA; 2 Department of Cancer Systems Imaging, The University of Texas MD Anderson Cancer Center, Houston, TX USA; 3 Department of Nuclear Medicine, The University of Texas MD Anderson Cancer Center, Houston, TX USA; 4 Present Address: RADIATE Theranostics R&D Platform, Therapeutics Discovery Division, The University of Texas MD Anderson Cancer Center, Houston, TX USA; 5 Present Address: Biomathematics & Bioinformatics Group, Indivumed, Falkenried 88/Bldg. D, 20251 Hamburg, Germany

**Keywords:** 2-[18F]BPA, Radiosynthesis, Boron neutron capture therapy, Copper-mediated fluorination, PET tracer

## Abstract

**Background:**

Boron neutron capture therapy relies on the selective accumulation of boron-containing compounds in tumor tissue, making accurate quantification of boron distribution essential for effective treatment planning. The amino acid analog boronophenylalanine is widely used as a boron delivery agent, yet direct assessment of its biodistribution remains challenging. A fluorine-18 labeled analog, 2-fluoro-boronophenylalanine, offers the potential to visualize and quantify uptake through positron emission tomography. However, reported radiosynthetic methods often suffer from low radiochemical yield, complex workflows, and limited compatibility with automated production platforms. The aim of this study was to design a stable precursor suitable for nucleophilic fluorination, develop a fully automated single-reactor radiosynthesis, and characterize the resulting tracer to support both preclinical use and future clinical translation.

**Results:**

A rationally protected precursor incorporating tert-butyloxycarbonyl and pinacol ester groups was synthesized and isolated with high chemical and enantiomeric purity. Using this precursor, an automated single-pot radiosynthesis was implemented on a commercial synthesis module employing copper-mediated nucleophilic fluorination followed by acidic hydrolysis. Across eight production runs, the method yielded 2-fluoro-boronophenylalanine with non-decay-corrected radiochemical yields of 3–5% and a total synthesis time of approximately 60–70 min. Radiochemical purity consistently exceeded 98%, and the molar activity at the end of synthesis ranged from 85 to 120 GBq per micromole. The final formulation remained chemically and radiochemically stable for at least four hours at room temperature. Analytical and chiral chromatographic assessments confirmed product identity, purity, and retention of stereochemical configuration.

**Conclusions:**

This study establishes a practical and fully automated radiosynthetic approach for producing 2-fluoro-boronophenylalanine using a single-reactor nucleophilic fluorination strategy. The method overcomes key limitations of electrophilic fluorination and multi-pot workflows, provides high radiochemical purity and suitable molar activity, and is compatible with commercially available synthesis equipment. These features support routine preclinical application and position the method for future current good manufacturing practice adaptation to enable clinical use in boron neutron capture therapy planning.

**Supplementary Information:**

The online version contains supplementary material available at 10.1186/s41181-026-00436-0.

## Background

Boron neutron capture therapy (BNCT) is a binary therapeutic strategy that relies on the selective accumulation of boron-containing compounds in tumor cells, followed by targeted irradiation with low-energy neutrons (Nedunchezhian et al. 2016). Among boron delivery agents, p-boronophenylalanine (BPA) has demonstrated clinical relevance due to its preferential uptake in malignant cells (Mishima et al. 1989). However, the success of BNCT depends critically on the ability to quantify BPA biodistribution in patients prior to treatment.

Positron emission tomography (PET) with a fluorine-18 labeled analog, 2-[^18^F]BPA, offers a powerful tool to support patient selection, treatment planning, and therapy monitoring (Ishiwata 2019). Despite this potential, existing radiosynthetic methods for 2-[^18^F]BPA are limited by poor yields, low molar activity, and workflows that are often incompatible with clinical good manufacturing practice (cGMP). Several reported approaches rely on electrophilic fluorination (Mairinger et al. 2015, Chang et al. 2023, Naka et al. 2022), which is low-yielding and operationally challenging.

Another study (He, et al., 2021, Ishiwata, 2019) described automated nucleophilic fluorination using a two-pot strategy, which limits its reproducibility on the more common single-reactor automated synthesis modules.

**Fig. 1:**
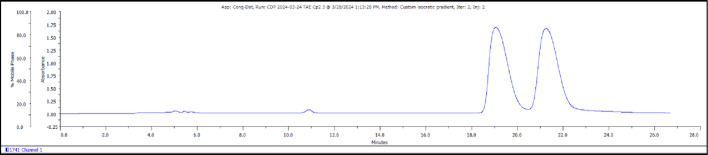
Preparative HPLC chromatogram showing the separation of 4a (18.5 min) and 4b (21 min)

**Fig. 2:**
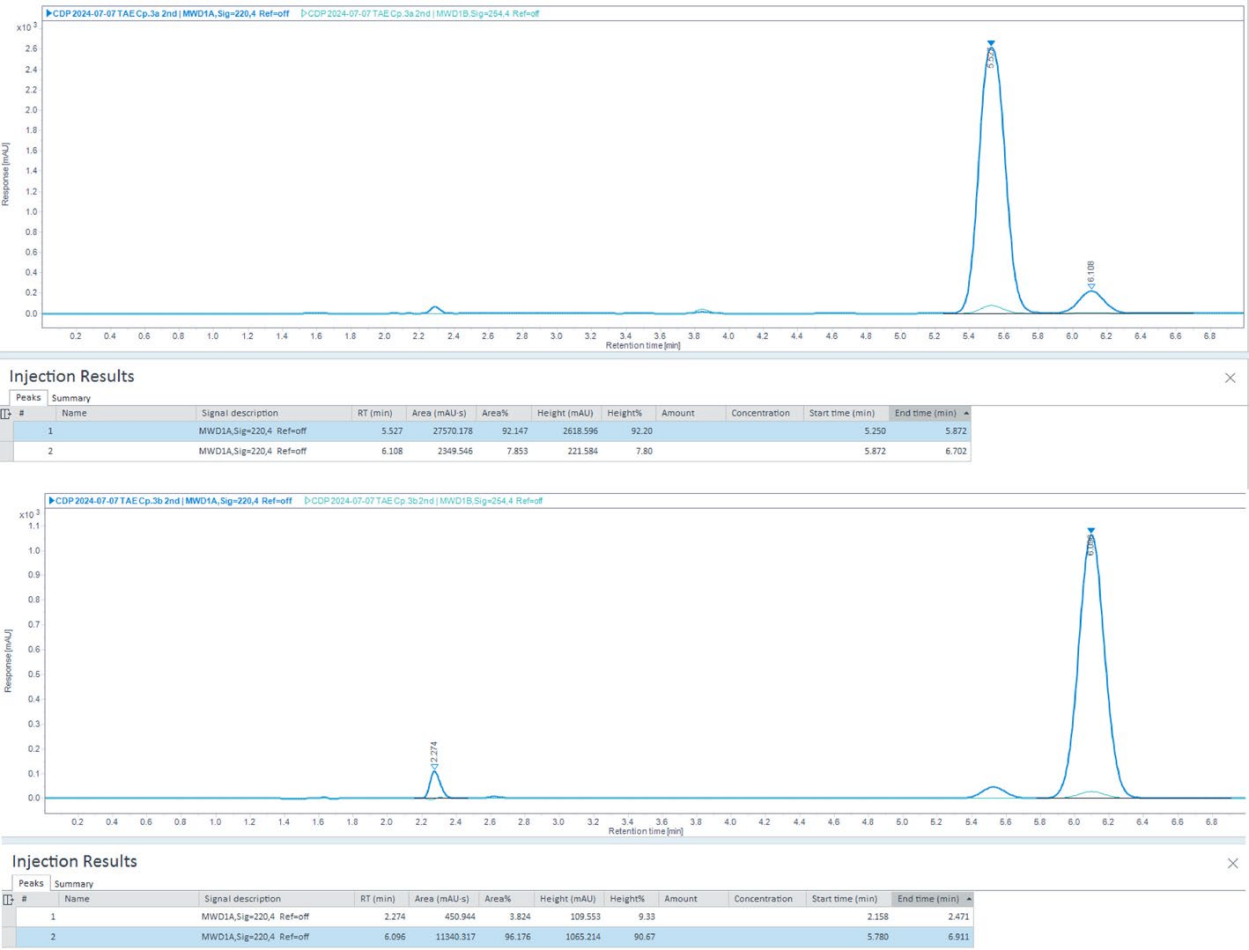
Chiral analytical HPLC chromatograms demonstrating the enantiomeric purity (enantiomeric excess) of intermediates **P2a** and **P2b**. In each trace, enantiomeric excess is assessed by the presence of a single dominant enantiomer peak and the minimal presence of the opposite enantiomer peak

To address these limitations, we sought to: (i) design and synthesize a rationally protected precursor for nucleophilic fluorination, (ii) develop a fully automated radiosynthetic method using copper-mediated fluorination on a commercial synthesis platform, and (iii) characterize the resulting radiotracer for radiochemical purity, molar activity, and stability to support preclinical applications and potential cGMP translation (Figs. 1, 2).

## Materials and methods

### General

**Fig. 3:**
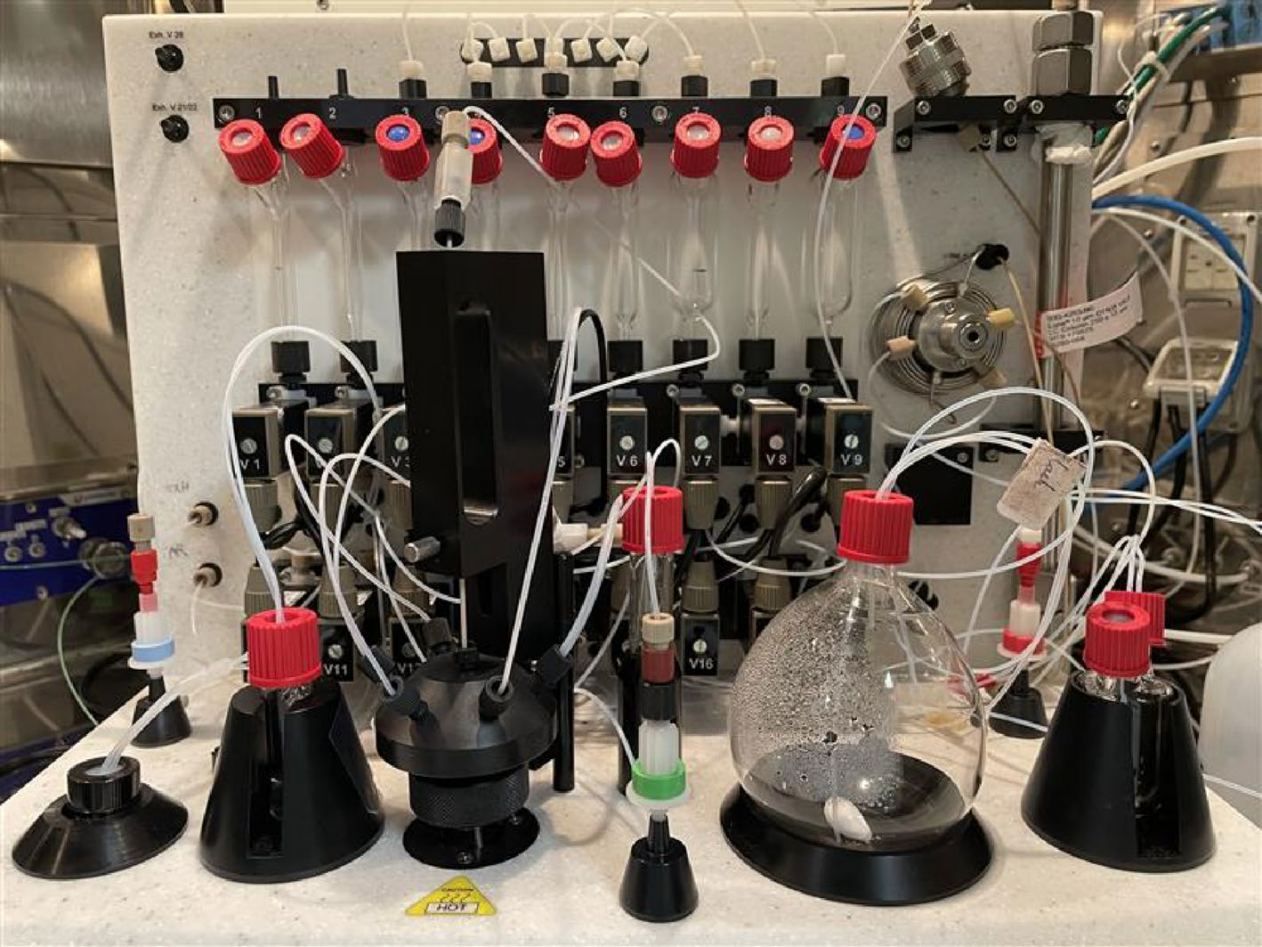
Photograph of the TRACERlab™ FXFN utilized for the synthesis of 2-[^18^F]BPA

The automated radiosynthesis of 2-[^18^F]BPA using GE TRACERlab™ FXFN (Figs. 2 and 3) was performed inside the lead-shielded COMECER hot cell. The Cryptand-222 (Kryptofix^®^ [2.2.2]) (Prod. # 800) and the preconditioned QMA light cartridges (Prod. # K-920) were purchased from ABX GmbH (Radeberg, Germany). Deionized water was obtained through a Milli-Q water (18 MΩ•cm) taken from a Millipore Milli-Q Integral 5 water purification system. All the other reagents were purchased from MilliporeSigma (St. Louis, MO). The ethanol 200 PROOF was obtained from Pharmco. Nitrogen and argon gas used primarily in drying and transferring of solutions were provided through Matheson Tri-gas. The automation synthesis on the TRACERlab™ FXFN module was controlled by the TRACERLab FX software. The Alumina N Plus Light Cartridge (Part # WAT023561) and tC18 Plus Short Cartridge (Part # WAT036810) were acquired through Waters (Milford, MA). Purification was performed using a Phenomenex Luna C18 column (250 mm Å~ 4.6 mm, 5 μm). The Kryptofix stock solution was prepared, in-house, with ratio of Cryptand-222 (10 mg/ml) and potassium carbonate (6 mg/ml) in methanol. The BPA precursor (Fig. 1) is produced in-house, as shown below. The reference standard (2 S)−2-amino-3-[4-(dihydroxyboranyl)−2-fluorophenyl]propanoic acid was purchased from Sigma Aldrich.

**Fig. 4:**
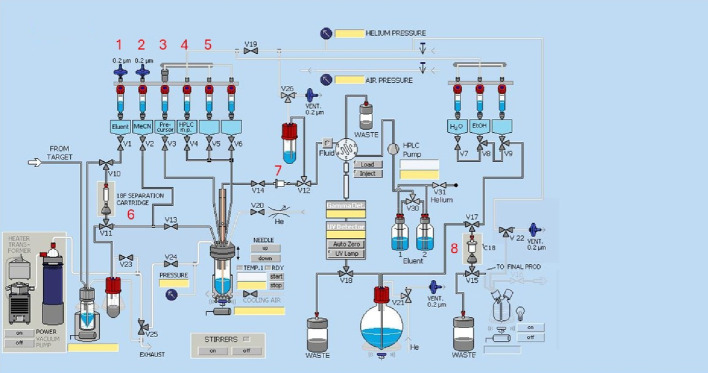
Schematic overview of the 2-[^18^F]BPA radiosynthesis on the GE TRACERlab™ FXFN module. The numbers denoted in RED represent the designation of item and position of reagents and consumables indicated in Table 1

**Table 1:** Material and reagent list used in the radiosynthesis of 2-[^18^F]BPA via TRACERlab™ FXFN module

Item #	Reagents or consumables
**1**	Potassium Carbonate/Kryptofix QMA Elution Solution,1.0 mL
**2**	Methanol, 0.6 mL
**3**	BPA precursor (10–15 mg), Cu(OTf)_2_Py_4_ (20 mg) dissolved in DMA/nBuOH/pyridine (800,100,100 µl), 1.0 mL
**4**	6 M HCl, 0.6 mL
**5**	HPLC MP, 1.5 mL
**6**	Pre-conditioned QMA light Sep-Pak cartridge, 1 cartridge
**7**	Alumina cartridge/Glass membrane filter assembly, 1 set
**8**	tC18 cartridge

### HPLC instrumentation

Semi-preparative HPLC purification was performed using a Phenomenex Luna C18 column (250 × 10 mm, 5 μm) with isocratic elution (0.1% acetic acid in water containing 1% methanol) at a flow rate of 4.0 mL/min. UV detection was set at 254 nm and radioactivity was monitored using an inline NaI(Tl) scintillation detector.

Analytical HPLC for quality control was performed on a Phenomenex Luna C18 column (250 × 4.6 mm, 5 μm) using 0.1% acetic acid in water/methanol (99:1, v/v) at a flow rate of 1.0 mL/min, with UV detection at 254 nm and a gamma detector for radioactivity.

Chiral HPLC purifications were performed on Gilson Preparative HPLC equipped with a Lux 5 μm Cellulose-1 (150 × 21.2 mm) column, using an isocratic gradient of 62% MeCN + 0.1% TFA (phase A) and 38% H_2_O + 0.1% TFA (phase B).

### Precursor synthesis overview

**Scheme 1:**
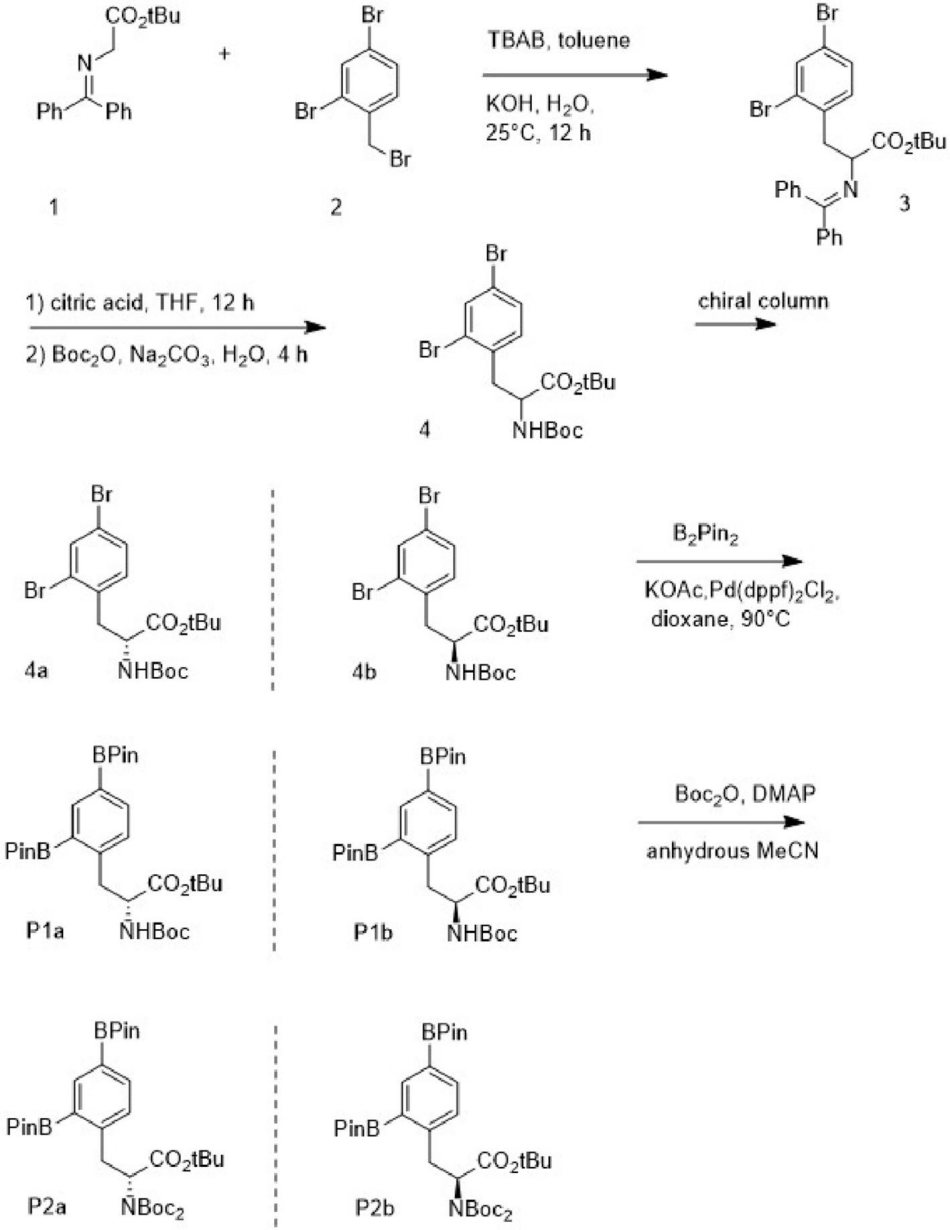
F-BPA precursor synthetic scheme

The precursor was synthesized through a multi-step route (Scheme 1) involving Boc protection of the amine group and pinacol ester formation of the boronic acid moiety to enhance stability during coupling and fluorination. This precursor was tailored for copper-mediated [^18^F]fluorination. Key intermediates were purified by column chromatography, and their identity confirmed by NMR and MS. Enantiomeric purity was assessed by chiral HPLC (Fig. 1).

### Procedure of preparation of intermediate 3

To a solution of N-(diphenylmethylene)glycerine tert-butyl ester 1 (4 g, 13.54 mmol), 2,4-dibromo-1-(bromomethyl)benzene 2 (4.45 g, 13.54 mmol) and tetrabutylammonium bromide (TBAB) (43.66 mg, 0.135 mmol) in 30 mL toluene, KOH (10 g, 0.178 mmol) in 8 mL H_2_O was added. Then the mixture was stirred at room temperature for 12 h and was monitored by thin layer chromatography (TLC) (hexanes: EtOAc = 20:1). After completion of the reaction, the mixture was diluted with 20 mL EtOAc and extracted with EtOAc (20 mL x 2). The combined organic layers were washed with brine (30 mL x 2), dried over anhydrous Na_2_SO_4_, filtered and concentrated under reduced pressure. The residue was purified by silica gel column chromatography (hexane/EtOAc = 100:1 to 20:1).

Yield: 3.64 g (91%).

¹H NMR (300 MHz, CDCl₃) δ 7.62 (m, 3 H), 7.35 (m, 7 H), 7.11 (d, J = 7.8 Hz, 1 H), 6.70 (d, J = 6.8 Hz, 2 H), 4.34 (dd, J = 9.5, 4.1 Hz, 1 H), 3.42 (dd, J = 13.4, 4.1 Hz, 1 H), 3.21 (dd, J = 13.6, 9.6 Hz, 1 H), 1.49 (s, 9 H).

MS (ESI⁺) m/z: [M + H]⁺ calculated 543.19, found 544.2.

### Procedure of preparation of intermediate 4

To a solution of compound 3 (1.2 g, 2.21 mmol) in 2.8 mL THF citric acid (1.27 g, 6.63 mmol) in 4.8 mL H_2_O was added and stirred for 12 h. Then Na_2_CO_3_ (1.17 g, 11.04 mmol) in 6 mL H_2_O and Boc_2_O (530.26 mg, 2.43 mmol) was added to the mixture and stirred for 4 h and is monitored by TLC (hexane: EtOAc = 10:1) until compound 3 was consumed completely. The reaction mixture was extracted with EtOAc (8 mL x 2). The combined organic layers were washed with (8 mL x 2), dried over anhydrous Na_2_SO_4_, filtered, and concentrated under reduced pressure. The residue was purified by silica gel column chromatography (hexane/EtOAc = 100:1 to 10:1).

Yield: 787.9 mg (74.4%).

¹H NMR (300 MHz, CDCl₃) δ 7.75 (d, J = 1.8 Hz, 1 H), 7.37 (dd, J = 8.2, 1.8 Hz, 1 H), 7.14 (d, J = 8.2 Hz, 1 H), 5.06 (d, J = 7.8 Hz, 1 H), 4.51 (m, 1 H), 3.24 (dd, J = 13.8, 5.9 Hz, 1 H), 3.01 (m, 1 H), 1.43 (s, 9 H), 1.38 (s, 9 H).

MS (ESI⁺) m/z: [M + H]⁺ calculated 479.09, found 480.1.

### Procedure of preparation of intermediate 4a and 4b

The compound 4 (787.9 mg, 0.747 mmol) was separated by multiple rounds of chiral chromatography (loading concentration 20–30 mg/ml) to obtain the enantiomers 4a and 4b. An isocratic gradient of 62% MeCN + 0.1% TFA (phase A) and 38% H_2_O + 0.1% TFA (phase B) was used as the mobile phase (Figs. 1 and 4). Compound 4a was eluted at −18.5–20 min and 4b at 21–22 min (flowrate 10 ml/min).

**Compound 4a**: Yield 48.1%. ¹H NMR (300 MHz, CDCl₃) δ 7.74 (d, J = 1.8 Hz, 1 H), 7.38 (dd, J = 8.2, 1.8 Hz, 1 H), 7.13 (d, J = 8.2 Hz, 1 H), 5.07 (d, J = 7.8 Hz, 1 H), 4.53 (m, 1 H), 3.25 (dd, J = 13.8, 5.9 Hz, 1 H), 3.02 (m, 1 H), 1.44 (s, 9 H), 1.39 (s, 9 H).

MS (ESI⁺) m/z: [M + H]⁺ calculated 479.09, found 480.1.

**Compound 4b**: Yield 45.8%.¹H NMR (300 MHz, CDCl₃) δ 7.73 (d, J = 1.8 Hz, 1 H), 7.39 (dd, J = 8.2, 1.8 Hz, 1 H), 7.14 (d, J = 8.2 Hz, 1 H), 5.06 (d, J = 7.8 Hz, 1 H), 4.53 (m, 1 H), 3.24 (dd, J = 13.8, 5.9 Hz, 1 H), 3.01 (m, 1 H), 1.44 (s, 9 H), 1.38 (s, 9 H).

MS (ESI⁺) m/z: [M + H]⁺ calculated 479.09, found 480.1.

Enantiomeric excess: 4a ~ 92%; 4b ~ 97%.

### Procedure of preparation of precursors P1a and P1b

A mixture of compound 4a (124 mg, 0.259 mmol), B_2_Pin_2_ (329 mg, 1.3 mmol), AcOK (101.74 mg, 1.04 mmol), Pd(dppf)Cl_2_ (18.91 mg, 0.026 µmol) in 1.5 mL dioxane was degassed and purged with Ar gas for 3 times. Then the mixture was stirred at 90 °C for 1 h under argon atmosphere. The crude reaction mixture was filtered and diluted with 14 mL H_2_O and extracted with EtOAc (7 mL x 3). The organic phase was washed with brine, dried over anhydrous Na_2_SO_4_, filtered and concentrated. The combined crude product was purified by preparative RP C18 HPLC with water and MeCN mixture as mobile phase. The precursor was also prepared using the same procedure from compound P1a: compound 4b (117 mg, 0.244 mmol), B_2_Pin_2_ (310.27 mg, 1.22 mmol), AcOK (95.93 mg, 0.977 mmol), Pd(dppf)Cl_2_ (17.83 mg, 0.024 µmol).

P1a yield: 106.9 mg (71%); P1b yield: 91.2 mg (67%).

#### P1a

¹H NMR (300 MHz, CDCl₃) δ 8.16 (s, 1 H), 7.76 (dd, J = 7.7, 1.4 Hz, 1 H), 7.22 (dd, J = 7.7 Hz, 1 H), 5.82 (d, J = 8.3 Hz, 1 H), 4.13 (m, 1 H), 3.13 (m, 2 H), 1.39 (s, 9 H), 1.30 (s, 12 H), 1.25 (s, 12 H), 1.19 (s, 9 H).

MS (ESI⁺) m/z: [M + Na]⁺ calculated 573.61, found 596.6.

#### P1b

¹H NMR (300 MHz, CDCl₃) δ 8.15 (s, 1 H), 7.75 (dd, J = 7.7, 1.4 Hz, 1 H), 7.21 (dd, J = 7.7 Hz, 1 H), 5.81 (d, J = 8.3 Hz, 1 H), 4.15 (m, 1 H), 3.13 (m, 2 H), 1.39 (s, 9 H), 1.31 (s, 12 H), 1.26 (s, 12 H), 1.20 (s, 9 H).

MS (ESI⁺) m/z: [M + Na]⁺ calculated 573.61, found 596.6.

### Procedure of preparation of the precursors P2a and P2b

A mixture of compound P1a (106.9 mg, 0.186 mmol), DMAP (24.83 mg, 0.203 mmol) and Boc_2_O (134.29 mg, 0.615 mmol) were dissolved in 30 mL anhydrous MeCN in a round-bottom flask stirred at room temperature for 24 h and monitored by TLC (hexane: EtOAc = 4:1) until the consumption of compound P1a was complete. The solvent of mixture was distilled under reduced pressure and purified with RP C18 HPLC (MeCN + 0.1% TFA 90–100%, H_2_O + TFA 0.1%, 10 − 0%, eluted at 16.7 min, flow rate 10 ml/min). The fraction of compound P2a and P2b were collected, neutralized with saturated aqueous Na_2_CO_3_, extracted with 2 × 50 ml dichloromethane, evaporated under reduced pressure, and repurified with silica gel chromatography (hexane: EtOAc = 4:1) to afford P2a 45 mg (36%). The compound P2b was also prepared using the same procedure from compound P1b: P1b (91.20 mg, 0.132 mmol), Boc_2_O (95.27 mg, 0.436 mmol), DMAP (17.61 mg, 0.144 mmol). P2b as the precursor with the naturally occurring (*S*)-configuration was used in the next step for radiofluorination. Figure 4 represents the chiral HPLC chromatograms showing enantiomeric excess of intermediates.

P2a yield: 45 mg (36%); P2b yield: 37 mg (31%).

#### P2a

¹H NMR (300 MHz, CDCl₃) δ 8.13 (s, 1 H), 7.67 (dd, J = 7.5, 1.2 Hz, 1 H), 6.96 (d, J = 7.6 Hz, 1 H), 5.15 (d, J = 3.8 Hz, 1 H), 3.89 (d, J = 3.9 Hz, 1 H), 3.06 (dd, J = 13.3, 11.4 Hz,2 H),1.42(s,9 H),1.27(s,42 H).

MS (ESI⁺) m/z: [M + Na]⁺ calculated 673.71, found 696.7.

#### P2b

¹H NMR (300 MHz, CDCl₃) δ 8.13 (s, 1 H), 7.67 (dd, J = 7.5, 1.2 Hz, 1 H), 6.97 (d, J = 7.6 Hz, 1 H), 5.13 (d, J = 3.8 Hz, 1 H), 3.89 (d, J = 3.9 Hz, 1 H), 3.07 (dd, J = 13.3, 11.4 Hz,2 H),1.41(s,9 H),1.26(s,42 H).

MS (ESI⁺) m/z: [M + Na]⁺ calculated 673.71, found 696.7.

Calculated m/z values are based on the assigned adduct ([M + H]⁺ or [M + Na]⁺).

### Automated radiosynthesis of 2-[^18^F]BPA

**Scheme 2:**
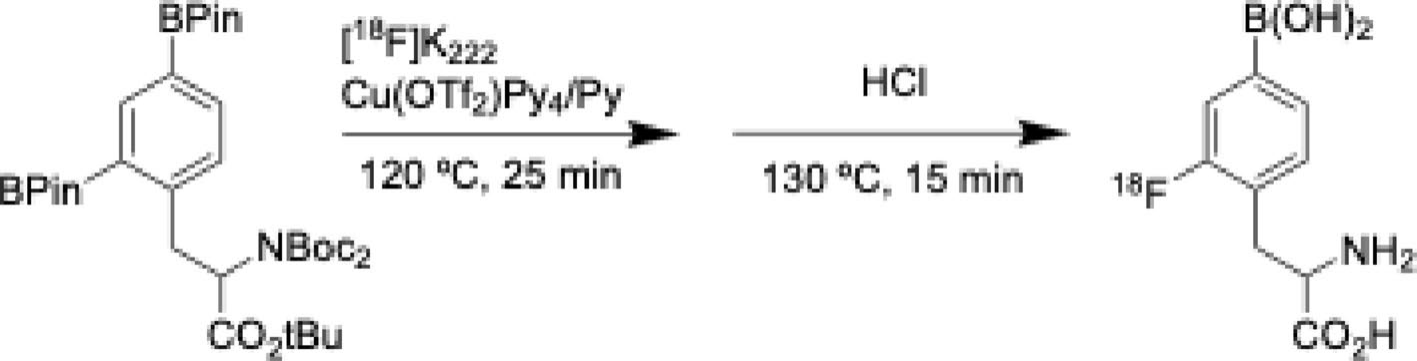
Radiolabeling scheme

The automatic radiosynthesis of 2-[^18^F]BPA is produced by copper-mediated nucleophilic substitution of aryl boronic ester precursor on a GE Tracerlab FX FN module, Scheme 2. The automatic radiosynthesis, including the nucleophilic radiolabeling of the precursor and the acidic hydrolysis of the radiolabeled intermediate, was performed with a single-pot reactor.

The list of the reagents and their corresponding vials in the Tracerlab are shown in Table 1.

The [^18^F]Fluoride was produced by irradiating 2.5 mL of enriched [^18^O]H_2_O with 60 µAh beam current from the 16.5 MeV GE PETrace cyclotron. The [^18^F]Fluoride was separated from the [^18^O]H_2_O and capture on the preconditioned QMA light cartridge then eluted with 1 ml of the Kryptofix solution. After drying the Kryptofix/[^18^F]KF solution by heating at 100 °C under vacuum and nitrogen gas flow, the precursor (15 mg) and Cu(OTf)_2_Py_4_ (20 mg) dissolved in DMA/nBuOH/pyridine (800,100,100 µl) were added to the reactor and heated at 120 °C for 25 min. After the completion of the labeling step, the reactor was cooled to 40 °C and 0.6 mL of 6 M HCl was added to the reaction mixture. Hydrolysis occurs as the reactor is heated at 130 °C for 15 min. Prior to semi-preparative HPLC purification, the crude reaction mixture was passed through an inline alumina cartridge/glass membrane filter assembly (Item #7, Table 1) to remove unreacted fluoride, particulate matter and residual copper species and to protect the HPLC column.

**Fig. 5:**
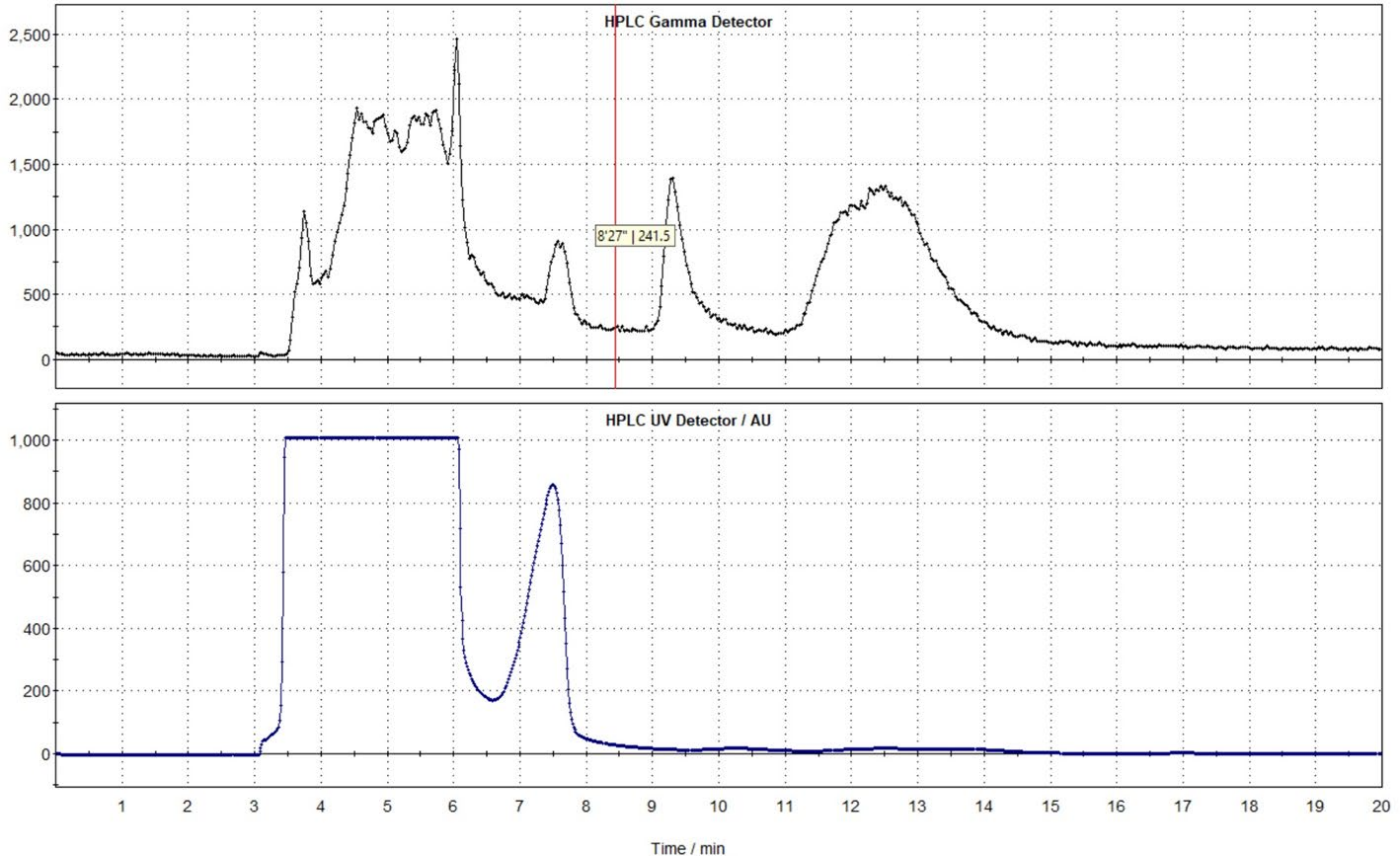
Semi-preparative HPLC radiochromatogram showing isolation of 2-[^18^F]BPA. The top portion shows the gamma signal, the bottom shows the correspondent UV signal. HPLC conditions: Phenomenex Luna C18 column (250 × 10 mm, 5 μm), mobile phase 0.1% acetic acid in water/methanol (99:1, v/v), flow rate of 1.0 mL/min, UV wavelength 254 nm

Purification was achieved using semi-preparative HPLC (Fig. 5) with isocratic elution (0.1% acetic acid/1% methanol, 4 mL/min). The desired fraction was collected, neutralized with NaHCO_3_, and passed through a tC18 cartridge. The product was eluted using 1 ml of ethanol and reformulated in 0.9% saline.

### Quality control

**Fig. 6:**
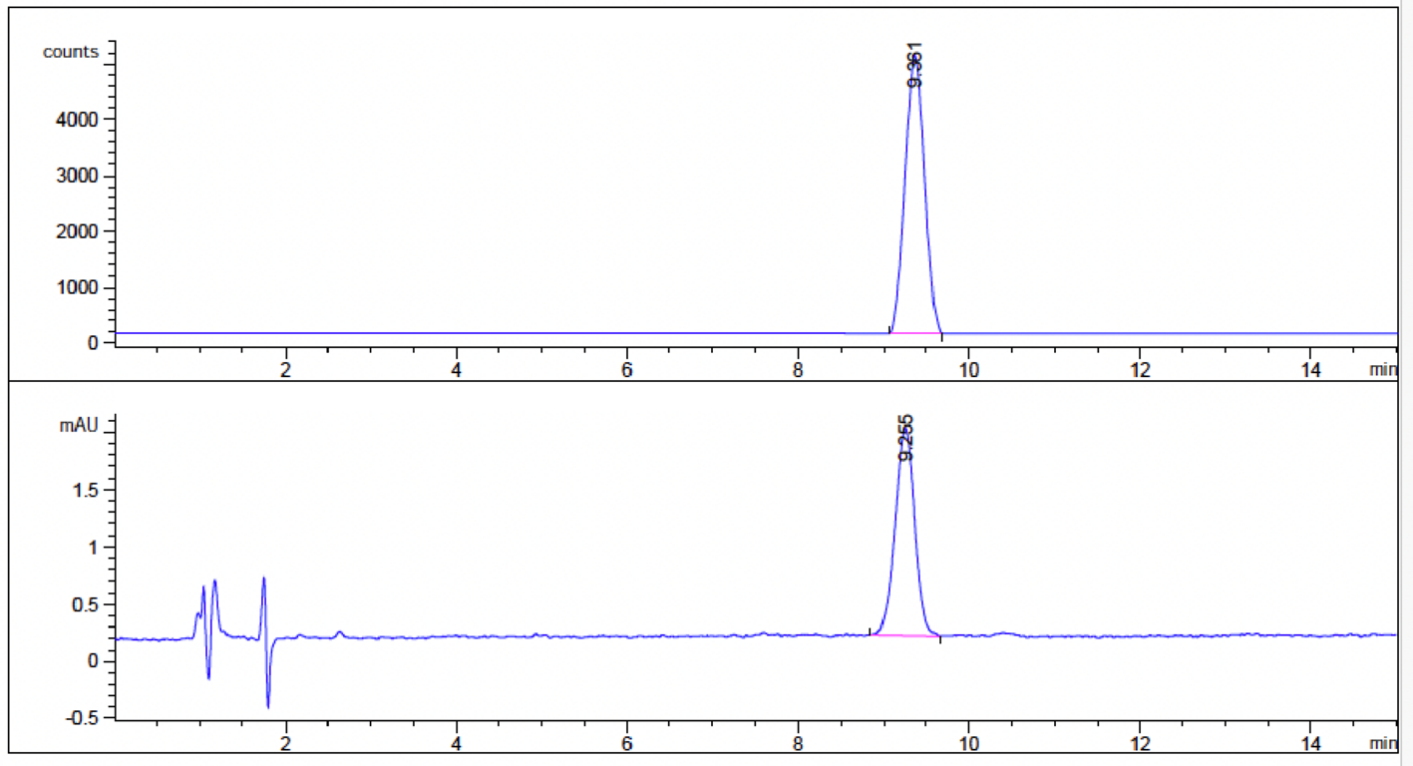
Analytical HPLC chromatogram of the final product ^18^F-BPA co-injected with the correspondent reference standard. HPLC conditions: Phenomenex Luna C18 column (250 × 4.6 mm, 5 μm), mobile phase 0.1% acetic acid in water/methanol (99:1, v/v), flow rate of 1.0 mL/min, UV wavelength 254 nm

Radiochemical identity and purity were confirmed by analytical HPLC (Fig. 6) with co-injection of a commercially available non-radioactive reference standard.

Radiochemical purity was determined from the radiochromatogram and consistently exceeded 98%. Radiochemical identity was confirmed by matching retention time between the radioactive product and the co-injected non-radioactive reference standard.

Enantiomeric excess was determined using chiral HPLC. Stability was monitored by HPLC over 4 h post-synthesis at room temperature.

A non-radioactive 2-[¹⁹F]BPA reference standard was purchased and used for analytical HPLC co-injection to confirm radiochemical identity of the final product.

The use of volatile organic solvents during radiosynthesis and purification was limited to methanol, DMA, n-butanol, and pyridine. These solvents were removed during semi-preparative HPLC purification and final formulation. Formal residual solvent analysis was not performed for preclinical production; however, the synthesis workflow and formulation strategy are compatible with residual solvent testing in accordance with ICH Q3C guidelines for future clinical translation.

Copper content was not quantified for this tracer during preclinical production. Copper analysis will be implemented as part of future cGMP translation, consistent with established regulatory expectations for copper-mediated radiofluorination methods.

For preclinical production, sterility and endotoxin testing were not performed; however, the synthesis platform and quality control strategy are compatible with implementation of these tests under cGMP conditions.

## Results

The final precursor was obtained in high purity (> 95%) as determined by 1 H NMR. Enantiomeric excess of the intermediates was 92% and 97%, respectively. Automated radiosynthesis consistently yielded 2-[^18^F]BPA in 3–5% non-decay-corrected radiochemical yield (NDCRY, *n* = 8) with a condensed total synthesis time of 60–70 min. Importantly, the fully automated workflow provides a shorter total synthesis time compared to several published procedures. Although the non–decay-corrected radiochemical yield is modest, it was reproducible across multiple production runs and sufficient for routine preclinical PET imaging when starting activities exceeded several tens of GBq.

Chiral HPLC analysis confirmed the enantiomeric purity of the radiolabeling precursor. Based on the use of an enantiomerically pure precursor and the absence of chemical transformations at the stereogenic center during radiofluorination and deprotection, retention of configuration in the final product is expected.

Radiochemical purity exceeded 98% in all batches, and molar activity ranged from 85 to 120 GBq/µmol at end of synthesis.

The final formulated product remained radiochemically stable for at least 4 h at room temperature, as assessed by analytical radio-HPLC.

Copper-mediated radiofluorination of aryl bis-boronic ester precursors is known to potentially afford multiple regioisomeric fluorinated products. Consistent with literature precedent, formation of minor regioisomeric radiolabeled byproducts cannot be excluded. In the present work, the desired 2-[¹⁸F]BPA was isolated by semi-preparative HPLC, and product identity was defined by analytical radio-HPLC co-injection with an authentic reference standard.

The results of the radiolabeling tests are shown in Table 2.

**Table 2:** Summary of radiochemistry production runs, including the non-decay corrected radiochemical yield (NDCRY)

Experiment	Initial Activity (mBq)	Product (mBq)	NDCRY %
001	9990	370	3.7
002	4440	22.2	0.5
003	16,650	259	1.6
004	22,200	1209.9	5.5
005	25,900	925	3.6
006	31,450	1073	3.4
007	24,050	925	3.8
008	18,870	962	5.1

## Discussion

This work establishes a practical, automated radiosynthetic pathway for 2-[¹⁸F]BPA. Compared to electrophilic fluorination approaches (Mairinger et al., 2015; Ishiwata 2019), our method delivers superior radiochemical purity and acceptable molar activity, while avoiding the need for specialized electrophilic fluorine production.

2-[^18^F]BPA is a promising agent for boron neutron capture therapy (BNCT); however, its application has been limited by the complexity of its multi-step radiosynthesis. Previous methods relied on laborious, multi-step and multi-pot reactions involving the use of ¹⁸F₂ gas, which yielded low radiochemical product yields. These approaches pose significant challenges for routine clinical implementation, as most automated synthesis modules are designed with only a single reactor. More recently, Chang et al. demonstrated that 2-[¹⁸F]BPA could be produced with high radiochemical yield, but the process was performed manually and required multiple reaction vessels, complicating its clinical translation (Chang et al. 2023). In this study, we developed a robust, two-step, single-pot automated synthesis protocol for multi-dose production of 2-[¹⁸F]BPA using the GE TRACERlab™ FXFN radio synthesizer (Figs. 2 and 3), which is cGMP-enabled and suitable for future clinical applications. Although the non–decay-corrected radiochemical yield remains modest, it is comparable to values reported for electrophilic and nucleophilic approaches to 2-[¹⁸F]BPA synthesis. Importantly, the fully automated single-reactor workflow prioritizes robustness, reproducibility, and compatibility with standard synthesis modules, which are critical considerations for routine implementation, and it provides high molar activity compared to the electrophilic method.

The protected precursor was designed to balance chemical stability with compatibility for copper-mediated nucleophilic fluorination and can be synthesized using standard organic chemistry techniques. While precursor production was performed in-house for the present study, the synthetic route is amenable to scale-up and technology transfer, supporting potential availability for other centers as interest in 2-[¹⁸F]BPA imaging expands.

Another important consideration is the complexity of the analytical radio-HPLC profile, which demonstrates the presence of multiple radiolabeled byproducts and closely eluting impurities. This complexity makes purification challenging, as baseline separation from the desired 2-[¹⁸F]BPA peak is required to ensure radiochemical purity and identity. Optimization of the semi-preparative HPLC conditions was therefore critical to achieving consistent recovery of the target fraction. We found that careful control of mobile phase composition and isocratic conditions was essential to reproducibly isolate 2-[¹⁸F]BPA without significant product loss. These parameters will be especially important for future cGMP adaptation of the method, where batch-to-batch reproducibility is mandatory.

Importantly, the entire workflow is compatible with a commercial synthesis module, supporting potential cGMP implementation. The robustness of the procedure, demonstrated across multiple runs, indicates suitability for routine production.

The demonstrated short-term stability of the final formulation further supports its suitability for preclinical imaging studies and aligns with practical requirements for routine radiotracer use.

In the context of BNCT, 2-[¹⁸F]BPA PET has the potential to refine patient selection and dosimetry, ultimately improving therapeutic outcomes. Our results represent a critical step toward enabling such clinical applications.

### Tracerlab vs. Fastlab and rationale to optimize the single pot reaction

Compared to cassette-based systems such as the GE Fastlab, the GE Tracerlab FX series offers greater flexibility and control over the radiosynthetic process. The Tracerlab allows for direct modification of reaction parameters, tubing configuration, and reagent delivery sequences, enabling rapid optimization of labeling conditions and adaptation to novel tracers or non-standard chemistries. This open architecture is particularly advantageous during early-stage development, when synthetic steps may require iterative fine-tuning or non-routine manipulations. In contrast, cassette-based modules are designed primarily for standardized, routine production under GMP conditions. While they provide improved reproducibility, reduced risk of operator error, and simplified regulatory compliance, their fixed design limits the user’s ability to modify the process or incorporate custom reagents. Thus, the Tracerlab system offers a more versatile platform for research and development, whereas cassette-based modules such as the Fastlab are better suited for high-throughput clinical manufacturing. The TRACERlab FXFN platform offers flexibility advantageous during method development, whereas cassette-based systems such as Fastlab provide benefits for standardized clinical production. Although the present work was performed on a TRACERlab module, the underlying chemistry is expected to be transferable to cassette-based systems following further optimization, supporting future clinical translation.

## Conclusions

We report a robust, automated radiosynthetic method for 2-[^18^F]BPA using copper-mediated nucleophilic fluorination of a rationally designed precursor. The method circumvents the challenges of electrophilic fluorination, delivers high radiochemical purity, acceptable molar activity, and formulation stability, and is compatible with commercial synthesis platforms. These features position the tracer for preclinical evaluation and pave the way for cGMP-compliant production in support of BNCT clinical workflows.

## Supplementary Information

Supplementary Material 1

## Data Availability

All data generated or analyzed during this study are included in this manuscript.

## Abbreviations

Al₂O₃: Alumina
Boc: tert-Butyloxycarbonyl
BPA: Boronophenylalanine
BNCT: Boron neutron capture therapy
Bq: Becquerel
BPin: Pinacol boronic ester (boronate pinacol ester)
C18: Octadecylsilane (reversed-phase)
cGMP: Current good manufacturing practice
Cu(OTf)₂(py)₄: Tetrakis(pyridine)copper(II) triflate
DMAP: 4-Dimethylaminopyridine
DMA: N, N-Dimethylacetamide
EOS: End of synthesis
ee: Enantiomeric excess
EtOH: Ethanol
GBq: Gigabecquerel
HCl: Hydrochloric acid
HPLC: High-performance liquid chromatography
ICH: International Council for Harmonisation
K₂CO₃: Potassium carbonate
K222: Kryptofix^®^ 2.2.2 (cryptand-222)
MBq: Megabecquerel
MeOH: Methanol
NaI(Tl): Thallium-doped sodium iodide scintillation detector
nBuOH: n-Butanol
NMR: Nuclear magnetic resonance
OTf: Trifluoromethanesulfonate (triflate)
PET: Positron emission tomography
py: Pyridine
QMA: Quaternary methyl ammonium (anion-exchange cartridge)
QC: Quality control
RCP: Radiochemical purity
RCY: Radiochemical yield
TFA: Trifluoroacetic acid
TLC: Thin-layer chromatography
UV: Ultraviolet (detector)
v/v: Volume/volume
