# Supplementary material for: Automated radiosynthesis of 2-[18F]BPA for PET-based planning of boron neutron capture therapy (BNCT): rational precursor design, radiofluorination, and characterization of methodology
Source: EJNMMI Radiopharm Chem. 2026 Mar 5;11:28. doi: 10.1186/s41181-026-00436-0 (PMC13076846; doi:10.1186/s41181-026-00436-0)
Supplement: Supplementary file 1 — Supplementary Material 1 [file 41181_2026_436_MOESM1_ESM.docx]

Response to the Reviewers

We thank the Editor and the Reviewers for their careful evaluation of our manuscript and for their constructive comments. We have revised the manuscript accordingly. All changes were implemented conservatively to improve clarity, methodological transparency, and compliance with radiochemistry and EJNMMI standards. Detailed point-by-point responses are provided below, with references to the revised manuscript.

Reviewer #1

Comment a

Discuss plan to optimize precursor and standard production—availability for other centers, GMP strategy, and automation plans.

Response:

A short paragraph has been added to the Discussion addressing precursor design, scalability, and availability, and clarifying that while precursor production was performed in-house for this preclinical study, the synthetic route is compatible with scale-up and technology transfer. Compatibility of the radiosynthetic workflow with future cGMP adaptation is also discussed (Discussion, Page 16, Lines 353-358).

Comment b

Discussion needed: yield seems to be low.

Response:

The Discussion has been revised to contextualize the observed non–decay-corrected radiochemical yield (3–5%) relative to previously reported electrophilic and nucleophilic syntheses of 2-[¹⁸F]BPA. The revised text emphasizes reproducibility and suitability for routine preclinical PET imaging, without speculative optimization claims (Discussion, Page 16, Lines 343-348).

Comment c

Discussion needed: QC on copper analysis.

Response:

The Quality Control section now explicitly states that copper content was not quantified for this tracer during preclinical production. We further clarify that copper analysis will be incorporated as part of future cGMP translation, informed by prior experience with a closely related copper-mediated radiofluorination process developed in our laboratory (Materials and Methods – Quality Control, Page 14, Lines 293-295).

Comment d

Enantiomeric excess discussion.

Response:

The Results section has been revised to clarify that enantiomeric purity was experimentally confirmed for the radiolabeling precursor by chiral HPLC. We further state that retention of configuration in the final product is expected based on the use of an enantiomerically pure precursor and the absence of chemical transformations at the stereogenic center during radiofluorination and deprotection, while explicitly noting that direct chiral analysis of the radiolabeled compound was not performed (Results, Page 14-15, Lines 307-310).

Comment e

Bacterial endotoxin tests and/or sterility of the product.

Response:

We have clarified in the Quality Control section that sterility and bacterial endotoxin testing were not performed for preclinical production. We further state that the synthesis platform and quality control framework are compatible with implementation of these tests under cGMP conditions for future clinical translation (Materials and Methods – Quality Control, Page 14, Lines 296-298).

Comment f

Stability discussion.

Response:

The Results section now explicitly states that the final formulated product remained radiochemically stable for at least 4 hours at room temperature, as assessed by analytical radio-HPLC. This point is also briefly reinforced in the Discussion (Results, Page 15, Lines 316-317; Discussion, Page 17, Lines 374-373).

Comment g

Use the SI system to report all the radioactivity units.

Response:

All radioactivity units have been reviewed and standardized to SI units throughout the manuscript (global revision).

Comment h

Mention the QC details in all the figure legends.

Response:

Relevant figure legends have been revised to explicitly reference the corresponding quality control methods, including analytical radio-HPLC and identity confirmation where applicable (Figure legends 6 and 7).

Comment i

Expand the abbreviations in the synthetic schemes.

Response:

Abbreviations used in synthetic schemes and captions have been expanded or clarified to improve readability (Global revision).

Reviewer #2

Comment 1

Proofreading, isotope nomenclature, units, and formatting inconsistencies.

Response:

The manuscript has been carefully proofread. Typographical errors, chemical subscripts, isotope notation, unit usage, and NMR formatting have been corrected throughout the manuscript (global revision). A list of abbreviations has been added at the end of the manuscript.

Comment 2

HPLC systems should be homogeneously described in the General section.

Response:

A dedicated subsection describing semi-preparative and analyticalHPLC systems has been added to the Materials and Methods section, including columns, mobile phases, flow rates, and detection modalities (Materials and Methods – HPLC instrumentation, Page 6, Lines 102-111).

Comment 3

Clarification of cartridges and Item #7.

Response:

The role of inline cartridges and the ion-exchange/glass membrane filter assembly (Item #7) has been clarified in the radiosynthesis description, including its function prior to semi-preparative HPLC purification (Materials and Methods – Automated Radiosynthesis, Page 12, Lines 267-271).

Comment 4

Cold reference standard and co-injection data.

Response:

The manuscript now clarifies that a non-radioactive (S)-2-[¹⁹F]fluoro-boronophenylalanine reference standard was purchased and used for analytical HPLC co-injection to confirm radiochemical identity of the final product (Materials and Methods – Quality Control, Page 5, Lines 98-99 and figure 7).

Comment 5

Correction of Scheme 2.

Response:

Scheme 2 has been revised to depict radiofluorination of the correct precursor used in this study (Scheme 2, Page 28).

Comment 6

Clarification of Figure 2.

Response:

The figure 2 has been removed, as it is not considered relevant.

Comment 7

Regioselectivity of radiofluorination.

Response:

The Results section has been revised to clarify that copper-mediated radiofluorination of aryl bis-boronic ester precursors may afford regioisomeric fluorinated products, consistent with literature precedent. We further explain that the desired 2-[¹⁸F]BPA was isolated by semi-preparative HPLC and identified by analytical radio-HPLC co-injection with an authentic reference standard, without claiming intrinsic regioselectivity (Results, Page 15, Lines 318-323).

Comment 8

QC insufficiency, residual solvents, and analytical evidence.

Response:

The Quality Control section has been expanded to clarify radiochemical identity and purity determination by analytical radio-HPLC. We explicitly state that residual solvent analysis was not performed for preclinical production (Materials and Methods – Quality Control, Page–).

Comment 9

Tracerlab vs Fastlab comparison and transferability.

Response:

The Discussion section has been revised to clarify the respective roles of the TRACERlab FXFN and cassette-based platforms such as Fastlab, and to state that the underlying chemistry is expected to be transferable following further optimization (Discussion, Page 18, Lines 393-398).

Comment 10

Reference formatting.

Response:

All references have been reviewed and reformatted in accordance with EJNMMI editorial guidelines (Body of manuscript and References section).
